# Risk factors for postoperative late deterioration in patients with spinal dural arteriovenous fistulas

**DOI:** 10.3389/fneur.2024.1412237

**Published:** 2024-07-08

**Authors:** Yuanhong Ge, Qingjia Lai, Wei Guo, Xuejun Xu

**Affiliations:** ^1^Department of Neurosurgery, Chengdu Second People’s Hospital, Chengdu, China; ^2^Department of Rehabilitation, Care Alliance Rehabilitation Hospital of Chengdu, Chengdu, China; ^3^Department of Neurosurgery, Tangdu Hospital, Air Force Military Medical University, Xi’an, China

**Keywords:** spinal dural arteriovenous fistula (SDAVF), neurological dysfunction (NDF), late deterioration (LD), risk factors, treatment

## Abstract

**Background:**

Approximately 86% of patients with spinal dural arteriovenous fistulas (SDVAFs) exhibit clinical improvement after surgery. However, 12%-55.8% of these patients experience late deterioration (LD) after an initial period of improvement. The risk factors for LD remain unclear. The aim of this study was to explore the risk factors for LD in SDVAF patients.

**Methods:**

The clinical data of patients who were admitted to two tertiary hospitals between June 2014 and May 2022 were reviewed. Patients were divided into two groups: the LD group and the no LD group. The severity of neurological dysfunction (NDF) was evaluated using the Modified Aminoff and Logue Scale. Univariable and multivariable Cox regression analyses were performed.

**Results:**

A total of 105 eligible patients were enrolled, with a mean age of 57.55 ± 9.42 years. The LD group comprised 37 individuals, while the no LD group consisted of 68 individuals. According to the univariable analysis, preoperative NDF severity and treatment strategy were associated with the risk of LD. According to the multivariable analysis, patients who underwent microsurgery (MS) had a lower risk of LD than did those who underwent endovascular treatment (EVT; HR 0.197, 95% CI 0.085-0.457), and patients with severe NDF had a higher risk of LD than did those with mild NDF (HR 3.604, 95% CI 1.226-10.588), whereas the risk of LD in patients with moderate NDF was similar to that of patients with mild NDF (HR 1.352, 95% CI 0.519-3.524).

**Conclusion:**

EVT and severe preoperative NDF are independent risk factors for LD.

## 1 Introduction

A spinal dural arteriovenous fistula (SDAVF) is a vascular anomaly characterized by an aberrant connection between an artery and a vein within the spinal dura mater. This aberrant connection disrupts physiological blood flow within the spinal cord, leading to progressive neurological dysfunction (NDF) (1). The annual incidence of SDAVF has been reported to range from 0.5 to 1.0 per 100,000 people (1, 2). The main clinical manifestations of SDAVF include gait dysfunction, paresthesia, and urinary dysfunction. However, due to its insidious onset and nonspecific manifestations, the anomaly is frequently misdiagnosed or subject to delayed diagnosis and treatment (3). According to one study, the mean duration from symptom onset to diagnosis can extend up to 23 months (3). Delayed diagnosis and treatment imply a poor prognosis, emphasizing the importance of early detection and intervention (4, 5).

Currently, digital subtraction angiography is the gold standard for diagnosing SDAVF, and effective treatment modalities include microsurgery (MS) and endovascular treatment (EVT). Timely intervention is crucial for delaying further deterioration and facilitating progressive improvement in neurological function (6). A relevant study reported an improvement rate of 86% (7). However, neurological function is likely to worsen after an initial improvement period, commonly referred to as late deterioration (LD), with an incidence of 12%–55.8% (7–9).

Several studies have indicated a probable association between improvement in SDAVF patients and factors such as age, duration of symptoms (DoS), severity of NDF, and treatment strategy (4, 10–15). However, the risk factors for postoperative LD in SDAVF patients are not fully understood. We retrospectively analyzed the clinical data of patients who were treated at two tertiary hospitals and who experienced LD in the postoperative period to explore the risk factors for postoperative LD and to provide more information for the prevention and treatment of LD.

## 2 Methods

### 2.1 Patients

The clinical data of patients with SDAVF who were consecutively admitted to two tertiary hospitals (Tangdu Hospital, Air Force Military Medical University and Chengdu Second People’s Hospital) between June 2014 and May 2022 were retrospectively reviewed. The inclusion criteria were as follows: (1) a diagnosis of SDAVF confirmed by digital subtraction angiography; (2) symptoms associated with SDAVF; (3) underwent either MS or EVT, but those who underwent combination therapy (MS + EVT) were excluded; and (4) complete information available. The exclusion criteria were as follows: (1) followed up for less than 3 months, (2) under age 18 years, (3) asymptomatic and diagnosed accidentally, and (4) lacked sufficient data.

### 2.2 Data collection and definition

The following data were collected: demographic characteristics, clinical features, treatment strategy, postoperative anticoagulant therapy, operation-related complications, recurrence confirmed by digital subtraction angiography, and LD. The Modified Aminoff and Logue Scale (mALS) was used to evaluate the severity of NDF. The mALS included 2 items: gait score (range from 0 to 5) and urine score (range from 0 to 3; Table 1). A higher score indicates a greater degree of NDF. The total mALS score is equal to the sum of the gait and urine scores. According to the total score, NDF was categorized into 3 grades: mild (score 0–2), moderate (score 3–5), and severe (score 6–8).

**Table 1:** Functional status determined using the Modified Aminoff-Logue Scale.

Grade	Definition
**Gait**	
0	Normal
1	Leg weakness, abnormal gait or stance but no restrictions in activity
2	Restricted activity
3	Requires 1 cane for walking
4	Requires 2 canes, crutches, or walker for walking
5	Confined to a wheelchair
**Urine**	
0	Normal
1	Hesitancy, frequency, urgency
2	Occasional urinary incontinence or retention
3	Total incontinence or persistent retention

The duration of symptoms (DoS) was defined as the duration from symptom onset to surgery. Operation-related complications included wound infection, subcutaneous effusion, central nervous system infection, and operation-related deterioration (an increase of at least 1 point in the mALS score or significant worsening of paresthesia) within 2 weeks after the operation. LD was defined as an increase of at least 1 point in the mALS score or significant worsening of paresthesia beyond 3 months after operation (5, 7, 16, 17). Follow-up duration was defined as the interval from the operation to LD or the last follow-up.

### 2.3 Statistical analysis

All continuous data are presented as the median or mean ± standard deviation, and categorical data are presented as frequencies. A univariable Cox regression analysis was performed first, then a multivariable Cox regression analysis was performed to investigate the potential risk factors for variables with a *p*-value ≤ 0.1 in the univariable Cox regression analysis or those with a potential impact on the outcome based on the literature. An α ≤ 0.05 indicated significance, and *p*-values ≤ 0.05 were considered significant. All the statistical analyses were performed with SPSS software (version 25, IBM, Armonk, New York, United States).

## 3 Results

**Figure 1:**
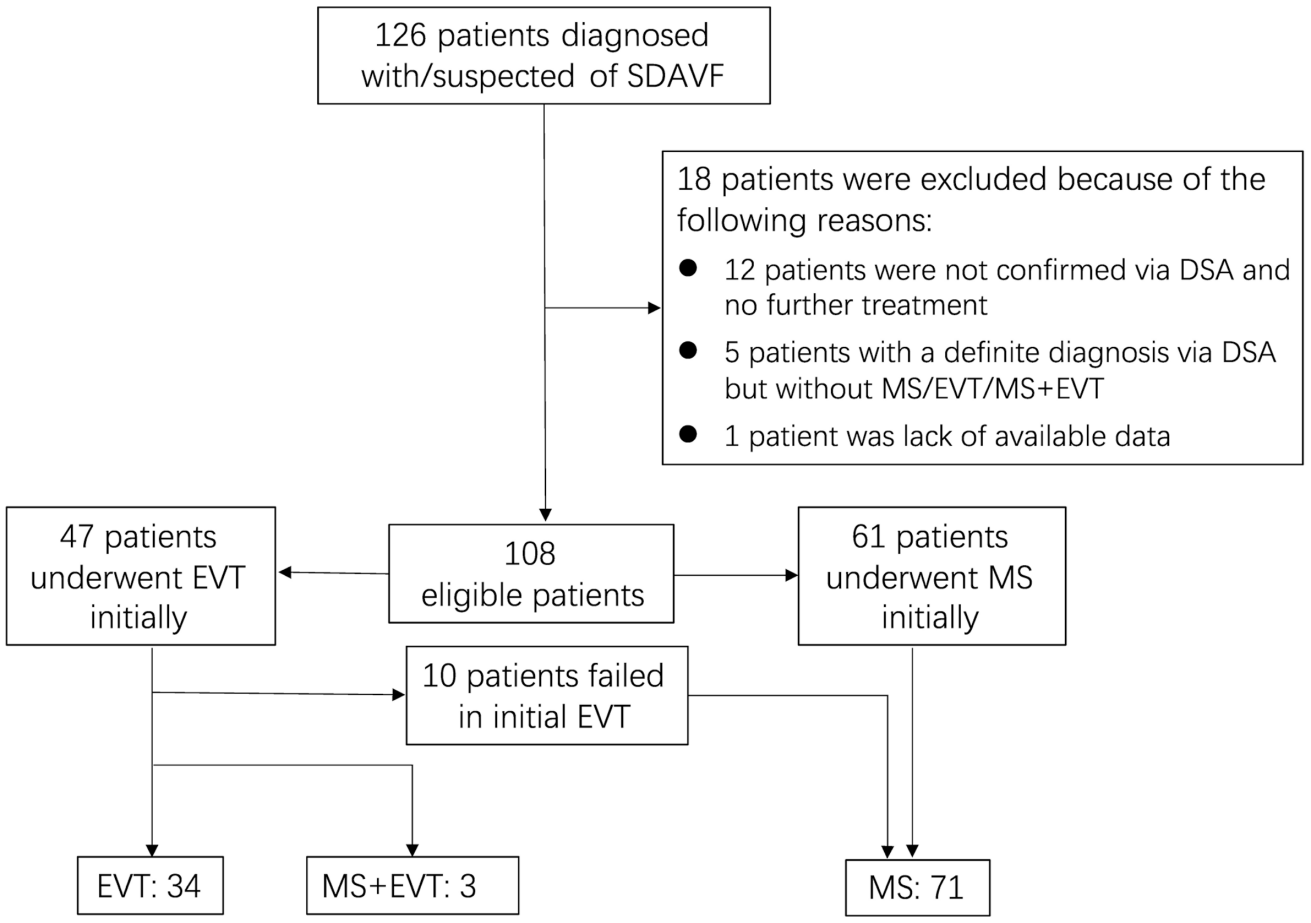
Flow diagram demonstrating inclusion and exclusion of screened patients. SDAVF, spinal dural arteriovenous fistula; DSA, digital subtraction angiography; MS, microsurgery; EVT, endovascular treatment.

In summary, a total of 126 patients with SDAVFs underwent screening, with 105 patients meeting the criteria (a detailed screening flowchart is shown in Figure 1). The male-to-female ratio was approximately 3.6:1 (82,23). The mean age of the patients was 57.55 ± 9.42 years (median, 57 years; range, 29–78 years), with 75.2% of patients aged between 50 and 70 years. The follow-up duration was 27.04 ± 13.15 months (median, 24 months; range, 6–88 months). A total of 82.9% of the fistulas were situated between the spinal segments of T5-L5. The mean DoS was 8.97 ± 10.05 months (median, 6 months; range, 0.03–72 months). The DoS was <6 months for 48 patients, 6–12 months for 20 patients, and ≥12 months for 37 patients. In addition, 42 patients had mild NDF, 38 had moderate NDF, and 25 had severe NDF. Thirteen patients received steroid therapy due to an initial misdiagnosis of idiopathic autoimmune inflammatory myelitis. Seventy-one patients underwent MS, and 34 patients underwent EVT. Twenty-four patients underwent postoperative anticoagulant therapy. Nine patients experienced operation-related complications, and 4 patients experienced radiological recurrence (arteriovenous fistula recanalization). All cases of recurrence were observed exclusively in patients who underwent EVT (Table 2).

**Table 2:** Detailed demographics and clinical characteristics.

	*N*	Percentage (%)
Age (≤57 yr)^*^	55	52.4%
Gender (Male)	82	78.1%
HBP	24	22.9%
DM	13	12.4%
**DoS (months)**		
<6	48	45.7%
6~12	20	19%
≥12	37	35.2%
**Pretreatment mALS (mean ± SD)**		
Gait	2.68 ± 1.64	/
Urine	0.99 ± 0.95	/
mALS in total	3.67 ± 2.18	/
Steroid	13	12.4%
**Severity of NDF**		
Mild	42	40%
Moderate	38	36.2%
Severe	25	23.8%
**Location of fistula**		
C	11	10.5%
T	64	61%
L	24	22.9%
S	6	5.7%
**Treatment**		
MS	71	67.6%
EVT	34	32.4%
Anticoagulation	24	22.9%
Complication	9	8.6%
Recurrence	4	3.8%
LD	37	35.2%
FU (mean ± SD, months)	27.04 ± 13.15	/

^*^The cutoff value refers to the median. HBP, high blood pressure; DM, diabetes mellitus; DoS, duration of symptoms; mALS, Modified Aminoff and Logue’s Scale; NDF, neurological dysfunction; MS, Microsurgery; EVT, endovascular treatment; LD, late deterioration; FU, follow-up; C, T, L, and S refers to Cervical, thoracic, lumbar, and sacral segments, respectively.

Thirty-seven individuals experienced LD, and 68 individuals did not. Among the patients who experienced LD, 18 were 57 years of age or younger, and 28 were males. Eight LD patients developed high blood pressure (HBP), while 4 developed diabetes mellitus (DM). Eight LD patients were inappropriately treated with steroids before the operation. Among the patients with LD, 16 patients had symptoms for less than 6 months, 7 had symptoms lasting between 6 and 12 months, and 14 had symptoms for 12 months or more. Among the patients who experienced LD, the preoperative severity of NDF was mild in 11 patients, moderate in 13 patients, and severe in 13 patients. The fistulas were primarily located in the thoracic segment. Twenty patients who experienced LD underwent MS, and 17 underwent EVT. Additionally, recurrence occurred in 4 patients with LD. The related demographic and clinical characteristics are summarized in Table 3.

**Table 3:** Univariable and multivariable cox regression analyses were applied to identify the risk factors of LD.

	LD (*N* = 37)	No LD (*N* = 68)	Univariable analysis		Multivariable analysis	
			HR (95%CI)	*p*	HR (95%CI)	*p*
Age (≤57 yr)^*^	18	37	1.025 (0.53, 1.985)	0.941	1.8 (0.767, 4.226)	0.177
Gender (Male)	28	54	0.528 (0.237, 1.177)	0.118	/	/
HBP	8	16	0.746 (0.325, 1.713)	0.489	/	/
DM	4	9	0.949 (0.331, 2.727)	0.923	/	/
DoS (months)				0.732		0.161
<6	16	32				
6~12	7	13	1.337 (0.537, 3.332)	0.532	2.458 (0.909, 6.651)	0.076
≥12	14	23	1.305 (0.615, 2.767)	0.488	1.837 (0.748, 4.511)	0.184
Steroid	8	5	1.528 (0.677, 3.449)	0.307	0.8 (0.312, 2.051)	0.642
Severity of NDF				0.018		0.042
Mild	11	31				
Moderate	13	25	1.149 (0.5, 2.64)	0.744	1.352 (0.519, 3.524)	0.537
Severe	13	12	3.036 (1.296, 7.112)	0.011	3.604 (1.226, 10.588)	0.020
Location of fistula				0.479		
C	2	9				
T	22	42	1.174 (0.273, 5.05)	0.830	/	/
L	11	13	2.05 (0.448, 9.379)	0.355	/	/
S	2	4	1.73 (0.241, 12.407)	0.586	/	/
Anticoagulation	11	13	1.042 (0.523, 2.075)	0.907	1.009 (0.451, 2.257)	0.982
MS	20	51	0.243 (0.117, 0.504)	< 0.001	0.197 (0.085, 0.457)	< 0.001
Complication	2	7	0.338 (0.079, 1.438)	0.142		

^*^The cutoff value refers to the median. HBP, high blood pressure; DM, diabetes mellitus; DoS, duration of symptoms; NDF, neurological dysfunction; MS, Microsurgery; LD, late deterioration; C, T, L, and S refers to Cervical, thoracic, lumbar, and sacral segments, respectively.

The Univariable Cox regression analysis revealed that both the preoperative severity of NDF and treatment strategy were associated with the risk of LD (*p* = 0.018 and < 0.001, respectively), while other factors (age, sex, HBP, DM, DoS, steroid therapy, NDF severity, fistula location, anticoagulant therapy, and complications) were not (Table 3). Considering that age, DoS, steroid therapy, and anticoagulant therapy could be potentially important risk factors, the multivariable Cox regression analysis included 6 variables (age, DoS, steroid therapy, anticoagulant therapy, NDF severity, and treatment strategy).

According to the multivariable analysis, only NDF severity and treatment strategy were significantly associated with the risk of LD (*p* = 0.042 and <0.0001, respectively). Patients who underwent MS had a lower risk of LD than those who underwent EVT (HR 0.197, 95% CI 0.085–0.457, *p* < 0.0001), and patients with severe NDF had a higher risk of LD than those with mild NDF (HR 3.604, 95% CI 1.226–10.588, *p* = 0.020); however, patients with moderate NDF had a similar risk of LD to those with mild NDF (HR 1.352, 95% CI 0.519–3.524, *p* = 0.537; Table 3).

Given that treatment modality was associated with the risk of LD, we performed a subgroup analysis to explore whether individualized treatment could help reduce the risk of LD (Table 4). The results showed that there was a lower risk of LD in patients who underwent MS when they had no HBP, no DM, a DoS < 6 months, a DoS ≥ 12 months, did not undergo steroid or anticoagulant therapy, or had a fistula in the thoracic segments (*p* < 0.05). For patients with moderate NDF, the difference in the risk of LD between patients who underwent MS and those who underwent EVT approached trend levels of significance (*p* = 0.084).

**Table 4:** Subgroup analysis comparing the differences between MS and EVT in reducing the risk of LD.

	LD (*n*, %)		HR (95% CI)	*p*
	MS	EVT		
overall	20 (28.2%)	17 (50%)	0.243 (0.117, 0.504)	<0.001
**Age** ^*^				
≤57 yr	8 (25%)	10 (43.5%)	0.185 (0.056, 0.608)	0.005
>57 yr	12 (30.8%)	7 (63.6%)	0.181 (0.063, 0.517)	0.001
**Sex**				
Male	14 (25.5%)	14 (51.9%)	0.196 (0.08, 0.481)	<0.001
Female	6 (37.5%)	3 (42.9%)	0.155 (0.025, 0.978)	0.047
**HBP**				
Yes	6 (30%)	2 (50%)	0.259 (0.047, 1.431)	0.121
No	14 (27.5%)	15 (50%)	0.207 (0.088, 0.487)	<0.001
**DM**				
Yes	2 (20%)	2 (66.7%)	0.722 (0.065, 7.963)	0.790
No	18 (29.5%)	15 (48.4%)	0.194 (0.086, 0.439)	<0.001
**DoS (months)**				
<6	8 (25.8%)	8 (47.1%)	0.302 (0.098, 0.933)	0.038
6~12	3 (20%)	4 (80%)	0.003 (0, 222.411)	0.304
≥12	9 (36%)	5 (41.7%)	0.231 (0.062, 0.869)	0.030
**Steroid**				
Yes	3 (42.9%)	5 (83.3%)	0.254 (0.048, 1.346)	0.107
No	17 (26.6%)	12 (42.9%)	0.25 (0.108, 0.581)	0.001
**Severity of NDF**				
Mild	4 (13.8%)	7 (53.8%)	0.001 (0, 60.082)	0.218
Moderate	8 (29.6%)	5 (45.5%)	0.304 (0.079, 1.172)	0.084
Severe	8 (53.3%)	5 (50%)	0.853 (0.274, 2.656)	0.784
**Location of fistula**				
T	14 (26.9%)	8 (66.7%)	0.268 (0.105, 0.689)	0.006
L	5 (45.5%)	6 (46.2%)	0.269 (0.053, 1.357)	0.112
Others^#^	1 (12.5%)	3 (33.3%)	0.014 (0, 156.019)	0.371
**Anticoagulation**				
Yes	11 (55%)	2 (50%)	0.555 (0.099, 3.109)	0.503
No	9 (17.6%)	15 (50%)	0.156 (0.06, 0.41)	<0.001

^*^The cutoff value refers to the median. ^#^Others refer to other segments, including thoracic and lumbar segments. HBP, high blood pressure; DM, diabetes mellitus; DoS, duration of symptoms; NDF, neurological dysfunction; MS, Microsurgery; EVT, endovascular treatment; LD, late deterioration; T and L refers to thoracic and lumbar segments respectively.

## 4 Discussion

The present study revealed that approximately three-quarters of the patients (75.2%) were aged between 50 and 70 years, most of whom were males, and fistulas were frequently located at T5-L5. These demographic characteristics were consistent with those of previous studies (16, 18, 19). Importantly, LD was associated with treatment modality and the severity of preoperative NDF but not with steroid use before surgery or anticoagulant therapy after surgery.

Both MS and EVT play crucial roles in preventing further deterioration and facilitating gradual improvement in neurological function. However, neurological function is likely to worsen after an initial period of improvement, which is commonly known as LD, with an incidence ranging from 12% to 55.8% (7–9). In this study, LD was observed in 35.2% of patients who were followed up for 27.04 ± 13.15 months. This deterioration rate was lower than the 55.8% reported in a recent study by Yang et al. (7), possibly due to the shorter mean follow-up duration in our study. The cause of LD is often unexplained, and the underlying mechanism is not well understood. First, arteriovenous fistula recanalization may lead to recurrence, subsequently causing LD. Additionally, impaired venous return capacity could affect the potential for spinal cord recovery, despite the improvement in venous hypertension after treatment (17). Finally, microcirculatory changes in the affected area after treatment may result in microthrombosis over time (20). Although recurrence may contribute to LD, the rate of LD attributed to recurrence is very low (17). Among the 37 patients who experienced LD, only 3 experienced LD due to recurrence (3/37, 8.1%). Therefore, recurrence is not a satisfactory explanation for LD. Durnford et al. (17) referred to LD not caused by recurrence as idiopathic or unexplained LD.

The risk of LD was higher in patients with severe NDF than in patients with mild NDF (HR 3.604, 95% CI 1.226–10.588, *p* = 0.020), but the risk was similar in those with moderate NDF (HR 1.352, 95% CI 0.519–3.524, *p* = 0.537). The increased risk of LD in patients with severe NDF may be attributable to a variety of factors. Due to occlusion of the fistula after the operation, the increased pressure of the draining vein decreases immediately, subsequently promoting neurologic improvement. However, irreversible impairment of venous drainage capacity may lead to persistent injury to the spinal cord (17). Severe damage to the spinal cord may result in greater impairment of the draining capacity of veins and worsening of nerve remodeling (17, 21).

Notably, MS was less likely to result in LD than was EVT (HR 0.197, 95% CI 0.085–0.457; *p* < 0.0001). Recanalization following embolization can partially explain this phenomenon (8, 17). Some scholars believe that recanalization after EVT is possible due to temporary occlusion of the fistula (22, 23). Recanalization was associated with recurrence. However, only 8.1% of patients with LD experienced recanalization in our study. We speculate that the embolic agent may induce the formation of microthrombi in the spinal cord, thereby increasing the risk of LD in EVT patients. Embolic agents have been shown to have toxic effects on nervous tissue (24–26). Therefore, it is speculated that the toxic effects of embolic agents on the delicate spinal cord may be another important reason why EVT patients are more susceptible to LD. On the one hand, controlling venous hypertension after EVT promotes the clinical symptom improvement. On the other hand, the inflammatory or foreign body reaction induced by the embolic agent may cause damage to the spinal cord. Initially, the former effect may outweigh the latter, but over time, the balance may shift.

Due to misdiagnosis, some patients with SDAVF may be inappropriately treated with steroids, which likely leads to acute clinical deterioration. This steroid-related deterioration was initially reported by Soderlund et al. (27). O’Keeffe (28) suggested that steroid-related deterioration could serve as a diagnostic indicator for SDAVF. Whereas Lee et al. (29) reported that the rate of steroid-related deterioration was 38%, a prospective study in 2020 showed that the incidence of steroid-related deterioration was as high as 77.4% (30). Some scholars have assumed that steroids increase the perfusion pressure of arteriovenous fistulas and increase the incidence of spinal venous thrombosis, subsequently affecting the microcirculation of the spinal cord and eventually resulting in acute clinical deterioration (30, 31). However, the effect of steroids on LD remains uncertain. In our study, there was no significant difference in the incidence of LD between patients who were treated with steroids and those who were not. This may partly reflect that the effects of steroids on neurological function are acute and temporary (29).

Given the potential for the induction of microthrombi in the spinal coronal venous plexus after surgery, which may subsequently result in clinical deterioration, postoperative anticoagulant treatment is considered beneficial (32, 33). Theoretically, anticoagulant therapy may reduce the risk of LD. However, similar conclusions were not drawn in our study. This negative conclusion was probably confounded by the duration of anticoagulant therapy and the target international normalized ratio (INR). There is currently no consensus on the optimal anticoagulant regimen (20). A case report by Knopman et al. (33) suggested that postoperative anticoagulant therapy for 6 months may be reasonable. Unfortunately, our data do not allow us to analyze the effect of treatment duration and INR on the incidence of LD.

One study suggested that patients with fistulas located below L4 were more likely to have LD (9), but we did not observe a similar result. Several previous studies have shown that age and DoS are associated with neurological recovery in SDAVF patients (4, 7, 13), while others have shown the opposite findings (11, 14). We did not observe an effect of age or DoS on the incidence of LD. Even though the DoS is not directly correlated with LD, a delayed diagnosis can lead to clinical deterioration, which has a direct correlation with LD. Therefore, it is of utmost importance to specify that SDAVF should always be included in the differential diagnosis of persistent and worsening leg weakness or pain, sensory disturbances, or sphincter dysfunction when no other clear etiology can be identified.

The above analysis showed that MS was less likely to lead to LD than EVT was. However, is MS consistently better than EVT in preventing LD across different subgroups? Or could EVT be more suitable for certain subgroups? Therefore, we conducted the subgroup analysis (Table 4). For patients with no HBP, no DM, a DoS < 6 months, a DoS ≥ 12 months, who did not undergo steroid treatment before surgery or anticoagulant therapy after surgery, or had a fistula in the thoracic segments, MS was more prone to reduce the risk of LD. For patients with moderate NDF, the difference in the risk of LD between patients who underwent MS and those who underwent EVT approached trend levels of significance (*p* = 0.084). However, for patients with mild or severe NDF, there were no significant differences. This finding contradicted our suspicion that patients with severe NDF are less likely to develop LD after undergoing MS than after undergoing EVT. It may be interpreted that severe NDF itself posed a high risk of LD, overshadowing the benefit of MS. These findings may help guide the choice of individualized treatment. Due to the small sample size, these findings should be interpreted carefully, and further validation is needed.

## 5 Conclusion

Postoperative LD is commonly observed in patients with SDAVFs. EVT and preoperative severe NDF are independent risk factors for LD. Future research on the mechanism of LD may help to provide very important information for preventing LD.

## Data availability statement

The original contributions presented in the study are included in the article/supplementary material, further inquiries can be directed to the corresponding authors.

## Ethics statement

Ethical approval was not required for the study involving humans in accordance with the local legislation and institutional requirements. Written informed consent to participate in this study was not required from the participants or the participants’ legal guardians/next of kin in accordance with the national legislation and the institutional requirements.

## Author contributions

YG: Writing – original draft, Formal analysis, Data curation. QL: Writing – original draft. WG: Writing – review & editing. XX: Writing – review & editing, Conceptualization.
